# Gene amplification during myogenic differentiation

**DOI:** 10.18632/oncotarget.6845

**Published:** 2016-01-08

**Authors:** Ulrike Fischer, Nicole Ludwig, Abdulrahman Raslan, Carola Meier, Eckart Meese

**Affiliations:** ^1^ Department of Human Genetics, Saarland University, 66421 Homburg/Saar, Germany; ^2^ Department of Anatomy and Cell Biology, Saarland University, 66421 Homburg/Saar, Germany

**Keywords:** myoblast, rereplication, gamma-H2AX, 53BP1, DSB

## Abstract

Gene amplifications are mostly an attribute of tumor cells and drug resistant cells. Recently, we provided evidence for gene amplifications during differentiation of human and mouse neural progenitor cells. Here, we report gene amplifications in differentiating mouse myoblasts (C2C12 cells) covering a period of 7 days including pre-fusion, fusion and post-fusion stages. After differentiation induction we found an increase in copy numbers of *CDK4* gene at day 3, of *NUP133* at days 4 and 7, and of *MYO18B* at day 4. The amplification process was accompanied by gamma-H2AX foci that are indicative of double stand breaks. Amplifications during the differentiating process were also found in primary human myoblasts with the gene *CDK4* and *NUP133* amplified both in human and mouse myoblasts. Amplifications of *NUP133* and *CDK4* were also identified *in vivo* on mouse transversal cryosections at stage E11.5. In the course of myoblast differentiation, we found amplifications in cytoplasm indicative of removal of amplified sequences from the nucleus. The data provide further evidence that amplification is a fundamental mechanism contributing to the differentiation process in mammalians.

## INTRODUCTION

For almost 30 years gene amplifications have been known in the development of amphibians and flies [1]. We recently reported gene amplifications during differentiation in human and mouse neural stem and progenitor cells [2, 3]. These studies raise the questions if gene amplification is a fundamental and widespread mechanism during the differentiation process. To further address this issue we searched for amplification during the differentiation of the skeletal muscle. Previously it was hypothesized that a strong increase of alpha-actin gene expression during chicken myoblast differentiation may result from a gene amplification mechanism [4]. Differentiation of skeletal muscle cells is a highly organized process with myoblasts differentiating from mononucleate cells to multinucleate myotubes and finally to mature myotubes. C2C12 cells derived from a mouse myoblast cell line [5, 6] were used in many studies that analyzed myogenic differentiation [7–9]. These cells differentiate rapidly, form contractile myotubes and produce characteristic muscle proteins. During early myogenesis there is evidence for double strand breaks of unknown origin in C2C12 cells [10]. Fork collapse at stalled replication forks play a role in the formation of double strand breaks [11] and both double strand breaks and fork collapse are likely accompanying the amplification process.

Here, we analyzed amplification during the differentiation of skeletal muscle. Our previous studies on gene amplification in non-malignant mammalian cells indicated that physiological amplification events may not only be restricted to small time windows but also to small number of cells. In the light of this finding, we used fluorescence *in situ* hybridization (FISH) to detect gene amplifications on single cell level. In addition, we used qPCR to monitor amplification over a time window of several days. The extended time window was selected to cover myogenic differentiation steps. A study from Hayward et al 1986 on primary chicken embryo myoblasts distinguished between prefusion stage (0-36h), fusion stage (48-72) and postfusion stage (more than 72h) [12]. In mouse C2C12 myoblasts maximal fusion is detectable between 24h and 36h and fusion is essentially completed after 72h to 96h [13]. In addition to the detection of amplification we searched for accompanying double strand break repair during myogenesis. We further set out to confirm our results on primary human myoblasts and *in vivo* on mouse cryosection.

## RESULTS

### Amplification of ACTA1, NUP133, MYO18B and CDK4 in single cells during mouse myogenesis

**Figure 1:**
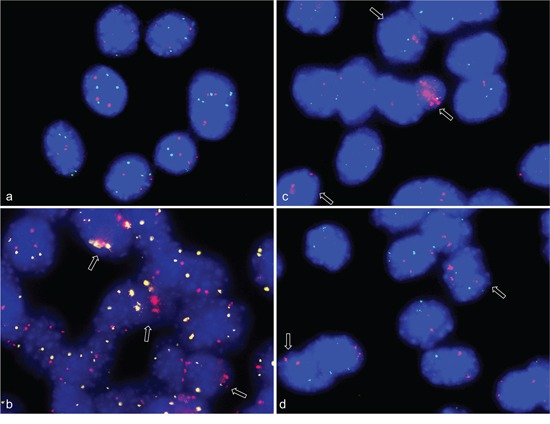
Gene amplifications on chromosomes 8qE2 and 5qF in differentiation induced C2C12 mouse myoblast cells FISH was used to analyze gene amplifications of two chromosomal loci (*MYO18B* in BAC RP23-6J9 and *ACTA1/NUP133* in BAC RP23-446H16) in nuclei from differentiation induced C2C12 mouse myoblast cells. In keeping with the known near tetraploid C2C12 karyotype, the undifferentiated C2C12 cells show tetraploid copy number for *MYO18B*(green) and triploid copy number for *ACTA1/NUP133* (pink) **a.** After four days of differentiation induction C2C12 cells show *MYO18B* (yellow) and *ACTA1/NUP133* (pink) gene amplification **b.** After 7 days of differentiation induction C2C12 cells show *ACTA1/NUP133* (pink) gene amplification and three to four signals for *MYO18B* (green) **c, d.** Representative cells with amplifications are marked by arrow. Nuclei were counterstained with DAPI.

**Figure 2:**
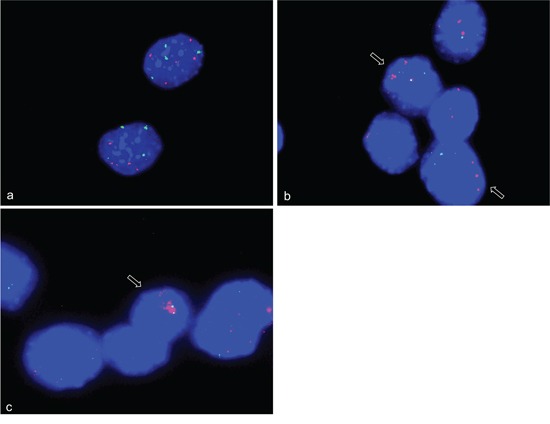
*CDK4* gene amplifications on chromosome 10qD3 in differentiation induced C2C12 myoblast cells FISH was used to analyze gene amplifications of *CDK4* (RP23-432F11) in nuclei from differentiation induced C2C12 mouse myoblast cells. *TERT* (RP23-132P5) was used as reference. Undifferentiated C2C12 cells show a tetraploid copy number for *TERT* (green) and five copies for *CDK4* (pink) **a.** After three days of differentiation induction C2C12 cells show CDK4 gene amplification (pink) and four signals for *TERT* **b, c.** Representative cells with amplifications are marked by arrow. Nuclei were counterstained with DAPI.

We analyzed C2C12 cells (ATCC), which represent a subclone generated from a mouse myoblast cell line [5, 6]. To search for gene amplification in single cells we used fluorescence *in situ* hybridization (FISH) on cells differentiating to myotubes over a period of seven days. We selected chromosome regions that harbor genes that were previously shown to be involved in myogenesis and/or to specifically show increased expression during myogenic differentiation. The chromosomal regions included 8qE2 containing *ACTA1*/*NUP133*, 5qF containing *MYO18B* and 10qD3 containing *CDK4*. It was previously shown that alpha-actin (*ACTA1*) and *MYO18B* expression increased during myogenic differentiation [13, 14]. *CDK4* was reported as amplified in tumors of myogenic origin [15]. In detail, the following BACs were used for FISH analysis: BAC RP23-446H16 containing genes *ACTA1* and *NUP133*, RP23-6J9 containing *MYO18B* and RP23-432F11 containing *CDK4*. In addition, as reference we used BAC RP23-132P5 that contained *TERT* which was previously not associated with myogenic processes. We define a copy number of the test gene as normal when both the number of signals corresponded to the genome ploidy and its fluorescence spot size equaled the spot size of the reference gene. An amplified copy number is defined by an increased signal number and/or by an increased fluorescence spot size of a test gene compared to the reference gene. FISH analysis on undifferentiated C2C12 cells revealed 3 signals for *ACTA1/NUP133*, 4 signals for *MYO18B*, 4-5 signals for *CDK4*, and 4 signals for *TERT* gene. Representative hybridization results of undifferentiated C2C12 nuclei are shown in Figures 1a and 2a. These results are consistent with the known near-tetraploid karyotype of C2C12 cells [16]. For amplification analysis we performed FISH on C2C12 cells at days 3-7 following differentiation inductions. The above time points were selected to span the mouse myoblasts fusion process that starts with the prefusion stage (0-36h), followed by the fusion stage (48-72), and that is completed after 72h to 96h [12, 13].

In the FISH analysis BAC *NUP133*/*ACTA1* identified a strong amplification in C2C12 myoblasts four days and seven days after induction of differentiation (Figure 1b–1d). Amplification of *MYO18B* was detected in C2C12 cells four days after differentiation induction (Figure 1b), and amplification of *CDK4* three days after differentiation induction (Figure 2b,c). Amplification frequencies of CDK4, NUP133, and MYO18B have been determined by analyzing 100 nuclei each (Table 1).

**Table 1:** Amplification frequencies from 100 nuclei analyzed by FISH

celltype	CDK4	NUP133	MYO18B	MDM2
C2C12 (mouse)	3-4%	5%	1-2%	n.a.
HSkM (human)	3%	1-10%	n.a.	5-10%

n.a.: not analyzed, variation depends on differentiation time

### Amplification of ACTA1, NUP133, MYO18B and CDK4 identified by qPCR during mouse myogenesis

**Figure 3:**
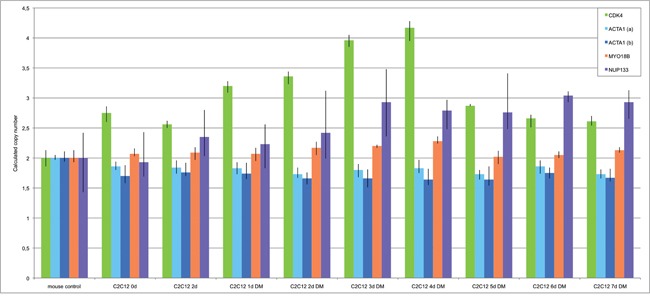
Amplification analysis of *CDK4, ACTA1, MYO18B* and *NUP133* in differentiation induced C2C12 myoblast cells using qPCR Amplification of *CDK4, ACTA1, MYO18B* and *NUP133* was analyzed by qPCR using TaqMan copy number assays. *TERT was used* as reference gene in the Taqman assays and mouse genomic DNA served as standard for normal diploid copy number. Copy numbers are shown as mean from four technical replicates with vertical lines indicating the range. Undifferentiated C2C12 cells were analyzed at two time points e.g. at time point zero and after two days in cell culture medium. Differentiation induced C2C12 cells were analyzed at seven time points after differentiation induction. These time points are indicated with DM (differentiation medium). *ACTA1* showed no amplifications in two independent TaqMan assays (light blue and dark blue). Likewise, *MYO18B* showed no amplifications (orange). *CDK4* showed a copy number gain after one day of differentiation induction and the highest copy number gain after 4 days of differentiation induction (green). *NUP133* showed a copy number gain at days 3, 4, 5, 6, and 7 after differentiation induction (purple).

After having identified gene amplifications in single cells, we used qPCR analysis (TaqMan) for the detection of amplification in DNA isolated from cells at two time points before differentiation induction (day zero and day 2) and seven time points after differentiation induction (1-7days) (Figure 3). The two time points before differentiation induction were included to identify possible gene amplification during normal cell culture i.e. cell culture without induction of differentiation. Since our FISH analysis showed amplifications only in smaller numbers of cells, qPCR, which analyzes all cells at each time point indicates such amplifications by a comparable small increase of copy numbers. There was a copy number increase of *CDK4* as compared to other genes prior to differentiation induction indicating an additional chromosome 10. This is consistent with our FISH results in undifferentiated cells that revealed 4-5 signals for *CDK4*. Following differentiation induction *CDK4* showed a constant increase in copy number from day 1 to day 4. At day 5 there was a decrease in copy number of *CDK4* back to the level that was found prior to the differentiation induction. A less pronounced but still detectable copy number increase was found for the gene *NUP133.* Here, the copy number increase was detectable after 2 days of differentiation induction and remained at moderate levels through days 3 to 7. PCR did not indicate copy number increase for *MYO18B*. While the FISH analysis detected a *MYO18B* amplification, the number of cells that harbor this amplification was apparently too low for a detection of an overall increased copy number by qPCR.

For *ACTA1* we found no evidence for amplification but a slight copy number decrease both in undifferentiated cells and throughout the differentiation process. This finding was confirmed by two *ACTA1* specific TaqMan assays. The reduced copy number likely reflects a lower chromosome 8q number, which was also indicated by three fluorescence signals per nucleus in FISH experiments as compared to the four fluorescence signals per nucleus in FISH experiments of the reference *TERT* on mouse chromosome 13qC1.

### Amplification of NUP133, CDK4 and MDM2 in primary human myoblasts during myogenesis

**Figure 4:**
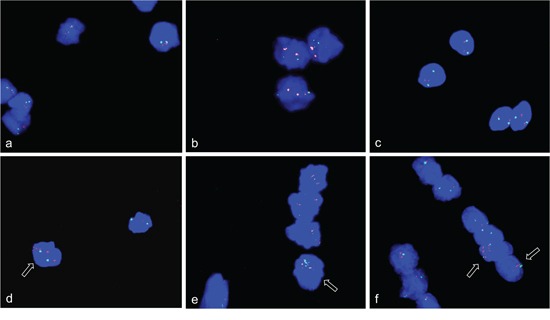
Gene amplifications on chromosomes 12q14-15 and 1q42.13 in differentiation induced HSkM human myoblast cells FISH was used to analyze *CDK4* (Rp11-571M6), *MDM2* (RP11-611O2) and *NUP133* (RP4-679K16) gene amplification in differentiation induced HSkM myoblast cells. Undifferentiated HSkM cells show two signals each for *CDK4* (pink) and the alpha centromere probe on chromosome 12 (green) **a,** three signals for the chromosome alphacentromere probe on chromosome 1 (red) and *NUP133* (green) in 50% of the cells indicating a gain of chromosome 1 **b,** and two signals each for *MDM2* (pink) and the centromere probe from chromosome 12 (green) **c.** HSkM cells show *CDK4* (pink) amplification after 2 days of differentiation induction **d.** *NUP133* (green) amplification after 3 days of differentiation induction **e,** and *MDM2* (pink) amplification after 2 days of differentiation induction **f.** Representative cells with amplifications are marked by arrow. Nuclei were counterstained with DAPI.

To confirm gene amplifications in an independent species we analyzed primary human myoblasts (HSkM; Invitrogen) by FISH. We used BAC RP4-679K16 that includes *NUP133* and *ACTA1* both of which are localized on human chromosome 1, BAC RP11-571M6 that includes *CDK4,* and BAC RP11-611O2 that includes *MDM2*. Both *CDK4* and *MDM2 are* localized on human chromosome 12. Alpha-centromere probes from chromosome 1 (D1Z5) and chromosome 12 (D12Z3) were used as references for non-amplified sequences. Representative hybridizations results for *NUP133*, *CDK4* and *MDM2* with corresponding centromere probe are shown in Figure 4a-c. We found trisomy for chromosome 1 in 50% of the undifferentiated primary myoblasts (4b). Amplifications of *CDK4* were detected after 2 days of differentiation induction (Figure 4d), amplifications of *NUP133* after 3 days of differentiation induction (Figure 4e), and amplifications of *MDM2* after 2 days of differentiation induction (Figure 4f). The amplification frequencies of CDK4, NUP133, and MDM2 have been determined by analyzing 100 nuclei each (Table 1).

### Amplification of NUP133, CDK4 and MDM2 identified by qPCR in primary human myoblasts during myogenesis

**Figure 5:**
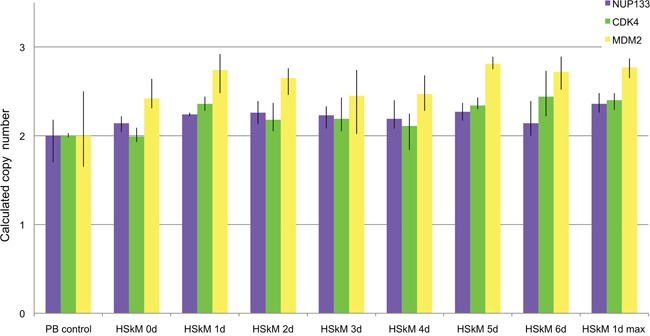
Amplification analysis of CDK4, MDM2 and NUP133 using qPCR in differentiation induced HSkM myoblast cells Amplification of *CDK4, MDM2* and *NUP133* was analyzed by qPCR using TaqMan copy number assays. *TERT was used* as reference gene in the Taqman assays and DNA from normal blood lymphocytes (PB) served as standard for normal diploid copy number. Copy numbers are shown as mean from four technical replicates with vertical lines indicating the range. Undifferentiated HSkM cells served as controls at day zero (0d). Differentiation induced HSkM cells were analyzed at six time points after differentiation induction. HSkM cells were also analyzed with a high seeding density after 1 day of differentiation induction (1dmax). *CDK4* showed the highest copy number after 6 days of differentiation induction (green). *NUP133* showed the highest copy number gain at day one after differentiation induction with cells seeded at high density (1dmax) (blue). *MDM2* showed copy number gains in undifferentiated HSkM cells and the highest copy number gain at days one, five and six each after differentiation induction (yellow).

We used qPCR analysis for amplification detection in DNA isolated from cells isolated at day zero and at six time points (1d, 2d, 3d, 4d, 5d, 6d) after differentiation induction. In addition, we investigated amplification in cells seeded at a high density after 1 day of differentiation induction. Cells seeded with high density are supposed to differentiate fast following differentiation induction. Normal blood lymphocytes (PB) served as control standard for a diploid karyotype. As indicated above, qPCR, which analyzes all cells at each time points indicates amplifications of few cells by a comparable small overall increase of copy numbers. A copy number increase was detectable for all genes (*NUP133, CDK4* and *MDM2*) after one day of differentiation induction (Figure 5). *NUP133* revealed the highest copy number increase after 1d of differentiation induction when seeded at high density. *CDK4* revealed a copy number increase after 1, 5 and 6 days. *MDM2* revealed the highest copy number increase of all genes tested.

### Cellular localization of amplified sequences by using combined FISH and immunofluorescence analysis during mouse myogenesis

**Figure 6:**
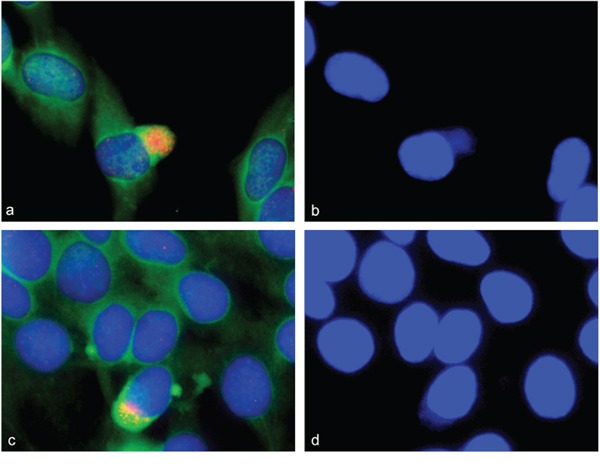
Gene amplification in alpha-actin expressing C2C12 cells Combined immunofluorescence and fluorescence *in situ* hybridization was used to confirm gene amplifications in differentiating myotubes. C2C12 cells stained positive for alpha-actin 4 days after differentiation induction (green). *NUP133* amplifications were found both inside and outside the nucleus **a, c.** Enhanced picture of a DAPI stain is shown to show DNA outside the nucleus **b, d.**

Since there is, as of yet, no evidence for persistent gene amplification in differentiated mammalian cells, the fate of amplifications that occur during differentiation remains to be elucidated. Using the combined analysis of alpha-actin as differentiation marker and interphase FISH of BACs for the above identified amplifications, we localized amplified sequences during myogenic differentiation. We found amplified gene signals not only within the nucleus but also amplifications that localized outside the nucleus. The signals in the cytoplasm that stained positive for alpha-actin were found for genes *ACTA1/NUP133* (Figure 6a and 6c). These cytoplasm signals were found specifically at days 3, 4, and 5 after differentiation induction. The presence of DNA outside the nucleus was confirmed by DAPI staining (Figure 6b and 6d). These data provide evidence that cells can eliminate amplifications from the nucleus during myogenesis.

**Figure 7:**
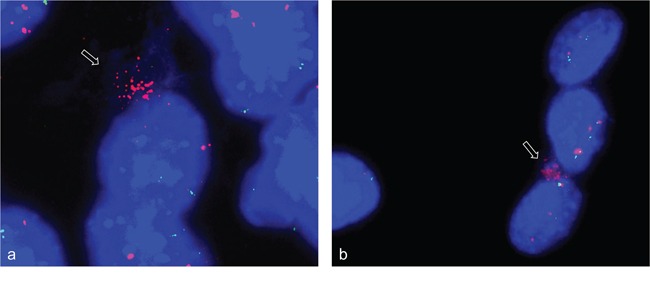
Gene amplifications outside the nucleus of C2C12 cells FISH was used to analyze *NUP133 and CDK4* gene amplification 3 and 5 days after differentiation induction. A cluster of strong fluorescence signals was detected for *NUP133* outside the nucleus of C2C12 cells at day five after differentiation induction (pink). **a.** Likewise a cluster of strong fluorescence signals was detected outside the nucleus for *CDK4* at day 3 after differentiation induction (pink) **b.** The signals indicate amplified *NUP133* and *CDK4* outside the nuclei. Weak and single signals indicating not-amplified *NUP133* and *CDK4* were detected in the nuclei. Weak and single signals were also found for *MYO18B* (green) (a) and *ANO4* (green) (b) both of which were not amplified. Nuclei were counterstained with DAPI.

Using simultaneous FISH with *ACTA1/NUP133*-BAC and *MYO18B*-BAC again revealed strong fluorescence signals for *ACTA1/NUP133* outside the nucleus (Figure 7a) while less pronounced signal for *ACTA1/NUP133 were found in the nucleus.* Likewise, strong fluorescence signals outside and weak signals inside the nucleus were found for *CDK4*. The signal of the reference probes *MYO18B* (green) (Figure 7a) and *ANO4* (green) (Figure 7b) were only found in the nucleus. Given that *ANO4* and *CDK4* were localized on the same chromosome, the finding of strong *CDK4* signals outside with *ANO4* signals remaining in the nucleus argues against a random exclusion of DNA material.

### Detection of double strand breaks, repair and single-stranded DNA during myogenic differentiation

**Figure 8:**
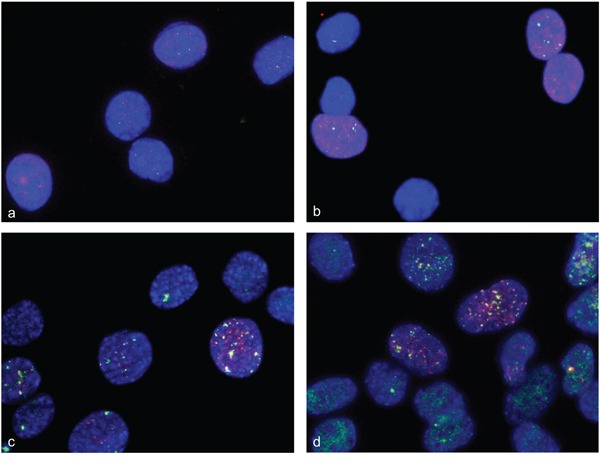
Double strand breaks and DNA repair in differentiation induced C2C12 cells Immunofluorescence assays were used to simultaneously analyze gamma-H2AX foci and 53BP1 protein in differentiation induced C2C12. Gamma-H2AX foci are shown in red fluorescence and 53BP1 protein specific foci are shown in green. Undifferentiated C2C12cells show a weak stain for gamma-H2AX and for 53BP1 **a, b.** After 3 days of differentiation induction, C2C12cells show both strong 53BP1 signals and gamma-H2AX foci, indicating double strand break repair **c, d.** Nuclei were counterstained with DAPI.

**Figure 9:**
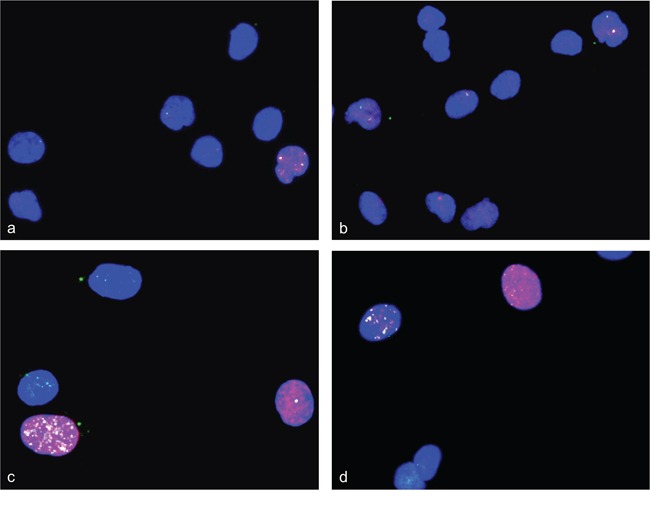
Double strand breaks and DNA repair in differentiation induced HSkM cells Immunofluorescence assays were used to simultaneously analyze gamma-H2AX foci and 53BP1 protein in differentiation induced HSkM cells. Gamma-H2AX foci are shown in red fluorescence and 53BP1 protein specific foci are shown in green fluorescence. Undifferentiated cells show a weak stain for gamma-H2AX and 53BP1 **a, b.** Two days after differentiation induction HSkM cells show both strong 53BP1 signals and gamma-H2AX foci indicating double strand break repair **c, d.** Nuclei were counterstained with DAPI.

There is evidence for double strand breaks detected by gamma-H2AX foci in C2C12 cells [10]. We detected gamma-H2AX foci in C2C12 at days 1, 2, 3, 4 and 5 after differentiation induction with a decrease in frequency after 4 days. Previous studies found disappearance of gamma-H2AX foci after 2 days [10]. Evidence for DNA repair in gamma-H2AX foci is indicated by localization of 53BP1 protein in those foci [17]. We found 53BP1-containing gamma-H2AX foci that are indicative for double strand break repair after 2, 3 and 5 days of differentiation induction in C2C12 cells. Representative immunofluorescence (IF) staining for gamma-H2AX and 53BP1 in undifferentiated C2C12 cells (a, b) and after 3 days of differentiation induction (c, d) are shown in Figure 8. Representative immunofluorescence staining for gamma-H2AX and 53BP1 in undifferentiated primary human myoblast cells (a,b) and after 2 days of differentiation induction (c,d) are shown in Figure 9. The detection of amplification and gamma-H2AX foci during myogenic differentiation is consistent with the hypothesis that gene amplification/rereplication might be responsible for double strand breaks, repair and replication stress.

### Correlation of gene amplification and double strand breaks

**Figure 10:**
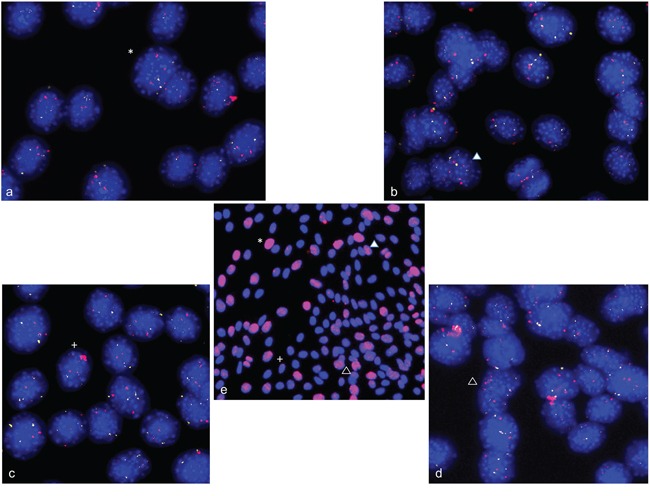
Double strand breaks and gene amplification in differentiation induced C2C12 cells Combined immunofluorescence assays and FISH were used to analyze the correlation between double strand breaks and amplification in C2C12 cells at day four after differentiation induction. Gamma-H2AX foci are shown in red in the overview picture (e) in the center of the figure. FISH analysis of *NUP133* (pink) and *CDK4* (white) is shown in the four outer pictures **a-d,** that represent enlarged portions of the overview picture with each position indicated by a separate symbol (cross, asterisk, filled and empty triangle). There is an overlap of cells with gamma-H2AX foci and cells with strong signals for *NUP133* (pink) and/or *CDK4* gene indicating amplifications of these genes. Nuclei were counterstained with DAPI.

Using a combined FISH-IF protocol, we analyzed whether C2C12 cells bearing gene amplifications also show an increased number of double strand breaks. C2C12 cells were differentiation induced for 4 days and first stained for gamma-H2AX. Subsequently, slides were used for FISH analysis with BAC-probes specific for *ACTA1/NUP133* and *CDK4*. Representative gamma-H2AX foci are shown in an overview picture with the results of FISH analysis shown in enlarged display windows. We found an overlap of cells with gamma-H2AX foci and cells with gene amplifications (Figure 10).

### *In vivo* analysis of gene amplification

**Figure 11:**
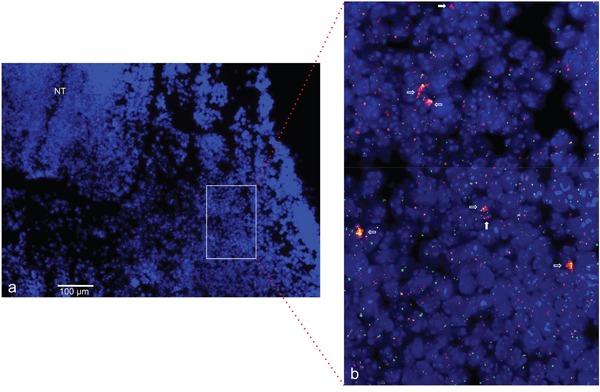
*CDK4* and *NUP133* amplification in mouse embryo stage E11.5 FISH was used to analyze *CDK4* and *NUP133* amplification *in vivo* on transversal cryosection from mouse abdominal region. The transversal cryosection is displayed as DAPI-stained overview with the neural tube indicated (NT) **a.** The results of FISH experiments are visible in the enlarged section **b.** Amplification of *CDK4* (pink) and/or NUP133 (yellow) is indicated by enlarged signals and/or multiple signals for CDK4 and NUP133. *TERT* was used as reference (green). Filled arrows indicate nuclei with *NUP133* amplifications and open arrows nuclei with co-amplifications of *NUP133* and *CDK4.* Individual cells with amplifications were detected in multiple regions of the developing myotome. Nuclei were counterstained with DAPI. Size calibration bar is 100μm.

To provide further evidence for a physiological role of gene amplification, we analyzed *ACTA1/NUP133* and *CDK4* for *in vivo* amplifications in mouse embryos. On the basis of dermomyotome development with myoblasts and early differentiating muscle cells, we selected embryonic stage E11.5 for the amplification analysis. Using FISH on cryosections from the mouse embryo we found evidence for an amplification of both *ACTA1/NUP133 and CDK4* in the dermomyotome (Figure 11).

## DISCUSSION

Gene amplification is well known as mechanism to elevate gene copy numbers during the development of amphibians and flies [1]. Recently we reported gene amplification during neural differentiation providing first evidence that gene amplification as a physiological mechanism is also established in mammals [2, 3]. Previous reports showed strongly increased alpha-actin gene expression during chicken myoblast differentiation [4]. Although the authors suggested gene amplification as likely cause for differential expression of alpha-actin gene during chicken myogenesis they could not reproducibly demonstrate amplification of alpha-actin gene during chicken myogenesis [12]. To follow up these findings, we analyzed gene amplification of alpha-actin (*ACTA1*) in myoblasts after induction of differentiation. For our FISH analysis we used a BAC probe for a genomic region that harbors the genes *ACTA1* and *NUP133*. While *NUP133* was confirmed to be amplified by using qPCR, qPCR failed to confirm the gene amplification of *ACTA1* that is localized 10 kb apart from *NUP133*. The increased gene expression of *ACTA1* during myoblast differentiation is likely due to other mechanisms than gene amplification. Nevertheless, our results confirmed the amplification of the genomic region including the genes *ACTA1* and *NUP133* during myogenic differentiation.

Since gene amplifications in mammals are largely known from tumors, it appeared legitimate to analyze genes, which are amplified in tumors for a possible amplification in a physiological context. Previous studies specifically suggested a relationship between gene amplification during tumor development and gene amplification during differentiation. We recently reported several genes that were amplified both in glioblastoma development and during neuronal progenitor cell differentiation [2]. A recent study reported *ERBB2* amplification during extravillous trophoblast differentiation and indicated a similar amplification event in the persistent gestational trophoblastic disease, which is a trophoblast-originating tumor [18, 19].

Here, we tested whether genes that were amplified in tumors of myogenic origin were also amplified during myogenic differentiation. *CDK4* and *MDM2* were amplified in leiomyosarcoma and rhabdomyosarcoma [15, 20]. We detected *CDK4* and *MDM2* amplification during myogenesis of human primary myoblast cells (HSkM) and *CDK4* amplification during myogenesis of mouse C2C12 cells. As for the scenario that may explain gene amplification both in the tumor and in the physiological context, stem cells or progenitor cells could possibly develop into tumor cells while maintaining their capacity for gene amplification. Alternatively, tumor cells may regain stem cell properties including the ability for gene amplification.

Regarding the mechanism of gene amplification, the process underlying the amplification in normal cells is likely different from the amplification mechanism known for mammalian tumor cells. The breakage-fusion-bridge-cycle model hypothesizes the collection of multiple copies by subsequent cycles in the course of cell divisions [21]. In differentiating cells the copy numbers are increased independent from cell division. The increase in normal cells may be due to multiple firing of replication origins and endocycling as reported for *Drosophila [22]*. One of the first models of gene amplifications that were hypothesized more than 30 years ago described an "onion-like" structure created by unscheduled re-replication in a replication bubble [23]. In our study, the clusters of frequently enlarged FISH signals in the nucleus suggest a re-replication mechanism. This hypothesis awaits further experimental confirmation.

Recent studies report massive genomic rearrangements in a single event in tumor cells referred to as chromothripsis [24]. This phenomenon is accompanied by DNA breaks, chromosome pulverization, and micronuclei formation [25, 26]. If and to what extend chromothripsis and gene amplification are related remains to be determined. Amplification may occur in the process of chromothripsis or alternatively chromothripsis may result from an unfinished and derailed amplification process at multiple replication origins in tumor cells. Although we have limited information on an overall genome involvement in the amplification process of differentiating myoblasts, we have evidence for a genome-wide amplification in normal neuronal progenitor cells. As of now, there is, however, no evidence for a role of chromothripsis in the amplification process in differentiating myoblasts.

Regarding the fate of amplified gene in physiological processes, amplified chorion DNA is removed by cell death of follicle cells in Drosophila. We did not find evidence for cell death as a general mechanism to remove sequences amplified during C2C12 myogenic differentiation. We found, however, evidence for the removal of amplified DNA from the nucleus. Using simultaneous FISH and IF we identified cells with amplified DNA not only in the nucleus but also in the cytoplasm of differentiation induced C2C12 cells. Specifically, we found intense fluorescence signals for *NUP133* and *CDK4* but not for the reference genes *ANO4* and *MYO18B* outside the nucleus, arguing for a selective exclusion of amplified sequences.

The determination of the general amplification frequency in differentiating cells remains challenging. First, at present the amplification frequency has been determined only for few genes; second, co-amplifications and heterogeneous cell population complicates an exact calculation. An indirect indication of amplification frequency can be made from cells with double strand breaks. After 4 days of differentiation induction 85% of cells revealed double strand breaks suggesting that these cells are involved in gene amplifications. We also clarify that this is a descriptive analysis and further functional proof is required to support a causative link between amplification and double strand breaks.

Genome wide DNA damage/strand breaks are known to occur during differentiation. Induced reduction of strand break formation leads to complete blockade of differentiation in myoblasts [10]. Our analysis confirmed the presence of double strand breaks (DSB) and provided evidence for DSB repair during myogenic differentiation. In addition, we were able to demonstrate that cells with gene amplifications simultaneously harbor DSBs. Our finding may also help to explain the persistence of amplifications in tumor cells. Tumor cells may have lost the stem cell ability to repair double strand breaks and the ability to exclude amplified DNA from the nucleus. Consistent with this idea is our finding, that genes that are amplified during developmental processes remain amplified in tumors. Examples include *CDK4*, which is amplified in neural progenitor cells and myoblasts during differentiation and *ERBB2,* which is amplified in differentiating trophoblasts. These genes were also amplified in tumors of the same origins including glioblastoma, leiomyosarcoma and trophoblast originating tumors. As for now, we are just at the very beginning of an understanding of the complex scenarios of gene amplification processes in a physiological context. A deeper insight into the mechanisms of gene amplification in differentiation processes will in turn help to shed light on the gene amplification mechanisms in tumor cells.

## MATERIALS AND METHODS

### Cell culture and differentiation

C2C12 (ATCC® CRL-1772^TM^) cells were grown in DMEM with 10% FCS. Differentiation was induced with change of the growth medium to DMEM supplemented with 2% horse serum. Human primary myoblast cells HSkM (Invitrogen) were grown in DMEM with 10% FCS. Differentiation was induced with change of the growth medium to DMEM supplemented with 2% horse serum. For immunofluorescence and for fluorescence *in situ* hybridization cells were grown on glass slides.

### qPCR analysis

TaqMan Copy Number Assays for genes *CDK4* (Mm00305758_cn)*, NUP133* (Mm00263211_cn), *ACTA1* (a: Mm00241646_cn; b: Mm00242070_cn) *and MYO18B* (Mm00167228_cn) were performed following manufacturers instructions. We used the *TERT* TaqMan Copy Number reference assay for relative quantitation of copy number of target genes. Mouse genomic DNA (Clontech) was used as control standard for normal diploid copy number.

TaqMan Copy Number Assays for genes *CDK4* (Hs00957586_cn), *NUP133* (Hs06590418_cn), and *MDM2* (Hs00181272_cn) were performed following manufacturers instructions. We used the *TERT* TaqMan Copy Number reference assay for relative quantitation of copy number of target genes. DNA from human normal blood lymphocytes (PB) was used as control standard for normal diploid copy number.

TaqMan assays were run in four technical replicates and results were analyzed using StepOne^TM^ Software v2.0 and CopyCaller^TM^ software.

### Fluorescence *in situ* hybridization

BAC clones were taken from the RP-11 (http://www.chori.org/bacpac/) libraries of the Welcome Trust Sanger Institute and available from SourceBioSciences, Germany. BAC-DNA (1μg) was either labeled with Alexa-488-dCTP (green fluorescence signals), with Alexa-555-dCTP (yellow fluorescence) or with Alexa-594-dCTP (pink fluorescence signals) using the FISHTag DNA labeling Kit according to the manufacturer's instructions. Differentially labeled probe DNAs (60 ng) were precipitated in the presence of mouse or human Cot-1 DNA. Samples were resuspended in hybridization mix (50% formamide, 2xSSPE, 10% dextrane sulphate and 4% SDS).

Differentiating C2C12 myoblast cells and control non-differentiating C2C12 myoblast cells were grown on glass slides and fixed in ice-cold methanol for 20 minutes. Human primary myoblast cells (HSkM) were treated alike. Slides were washed in PBS for 5 minutes and treated with 0.02% Tween-20 for 5 minutes. Slides were RNase treated (100 μg/ml RNaseA in 2x SSC) for 1h at 37°C and pepsin treated (0.005% in 0.01 M HCl at 37°C) for 10 minutes. Postfixation was performed using 1% formaldehyde/1x PBS for 10 minutes at room temperature. Hybridization and posthybridization washes were as described previously [3].

For simultaneous fluorescence *in situ* hybridization and immunofluorescence, differentiating C2C12 cells on glass slides were fixed in ice-cold methanol for 20 minutes and permeabilized with 0.2% Tween-20 for 5 minutes. Postfixation was done by 1% formaldehyde/1x PBS for 10 minutes at room temperature. Slides were blocked with goat serum, incubated for 1 h with rabbit antibody monoclonal against muscle-Actin (ab156302, Abcam) and detected using an Alexa-488 coupled secondary antibody (Invitrogen). Finally, slides were dehydrated by an ascending ethanol series (70%/80%/96%) and air-dried. Hybridization and post hybridization washes were as described above.

For immunofluorescence C2C12 cells or human primary myoblast cells (HSkM) were grown on glass slides, fixed and permeabilized as above and incubated with following antibodies: mouse antibody monoclonal against gamma-H2AX (ab26350, Abcam) and rabbit antibody polyclonal against 53BP1 (ab21083, Abcam). Detection was done with an Alexa-488 coupled secondary antibody against rabbit and Alexa-594 coupled secondary antibody against mouse (Invitrogen).

Fluorescence images were captured with an Olympus AX70 microscope using CellSens software from Olympus.

### Fluorescence *in situ* hybridization on mouse embryo cryosections

Transverse cryosections (10 μm) from mouse trunk at embryonic stage E11.5 were treated with Carnoy's fixative for 15 min at 4°C, pepsin-digested and fixed in 4% paraformaldehyde in diethylpyrocarbonate-treated PBS for 10 minutes. Probes were labeled as described above, applied to the section and denatured for 5 min at 80°C. Hybridization was maintained for 2 days at 37°C. Posthybridization washes and DAPI staining were as described above.
